# Development and validation of a simple tool composed of items on dyspnea, respiration rates, and C-reactive protein for pneumonia prediction among acute febrile respiratory illness patients in primary care settings

**DOI:** 10.1186/s12916-022-02552-5

**Published:** 2022-10-18

**Authors:** Fengming Ding, Lei Han, Dongning Yin, Yan Zhou, Yong Ji, Pengyu Zhang, Wensheng Wu, Jijing Chen, Zufang Wang, Xinxin Fan, Guoqing Zhang, Min Zhang

**Affiliations:** 1 Department of Respiratory and Critical Care Medicine, Shanghai General Hospital, Shanghai Jiao Tong University School of Medicine, Shanghai, China; 2 Department of Infectious Disease, Leishenshan Hospital, Wuhan, Hubei Province China; 3 Department of Infectious Disease, Shanghai General Hospital, Shanghai Jiao Tong University School of Medicine, Shanghai, China; 4 Department of Internal Medicine, People’s Hospital of Wannian County, Shangrao, Jiangxi Province China; 5 Department of Internal Medicine, Dongfang People’s Hospital, Dongfang, Hainan Province China; 6 Department of General Medicine, Zhongzhuang Town Health Center of Honghuagang District, Zunyi, Guizhou Province China; 7 Department of Tuberculosis, Fuzhou Pulmonary Hospital, Fuzhou, Fujian Province China; 8 Department of Respiratory Medicine, Jiangqiao Hospital of Jiading District, Shanghai, China

**Keywords:** Pneumonia, Prediction, Risk factor, Acute febrile respiratory illness

## Abstract

**Background:**

Acute febrile respiratory illness (AFRI) patients are susceptible to pneumonia and suffer from significant morbidity and mortality throughout the world. In primary care settings, the situation is worse. Limited by computerized tomography resources and physician experiences, AFRI patients in primary care settings may not be diagnosed appropriately, which would affect following treatment. In this study, we aimed to develop and validate a simple prediction model to help physicians quickly identify AFRI patients of pneumonia risk in primary care settings.

**Methods:**

A total of 1977 AFRI patients were enrolled at two fever clinics in Shanghai, China, and among them, 727 patients who underwent CT scans were included in the analysis. Acute alveolar or interstitial infiltrates found on CT images were diagnosed with pneumonia. Characteristics and blood parameters were compared between pneumonia and non-pneumonia patients. Then a multivariable model for pneumonia prediction was developed through logistic regression analysis. Its value for pneumonia prediction was prospectively assessed in an external multi-center population, which included 1299 AFRI patients in primary settings from 5 different provinces throughout China.

**Results:**

In the model development population, pneumonia patients (*n* = 227) had a longer duration of fever; higher frequencies of purulent sputum, dyspnea, and thoracic pain; and higher levels of respiration rates and C-reactive protein (CRP) than non-pneumonia patients (*n* = 500). Logistic regression analysis worked out a model composed of items on dyspnea, respiration rates > 20/min, and CRP > 20 mg/l (DRC) for pneumonia prediction with an area under curve (AUC) of 0.8506. In the external validation population, the predictive accuracy of the DRC model was the highest when choosing at least one positive item (1 score) as a cut-off point with a sensitivity of 87.0% and specificity of 80.5%. DRC scores increased with pneumonia severity and lung lobe involvement and showed good performance for both bacterial and viral pneumonia. For viral pneumonia, dyspnea plus respiration rates > 20/min had good predictive capacity regardless of CRP concentration.

**Conclusions:**

DRC model is a simple tool that predicts pneumonia among AFRI patients, which would help physicians utilize medical resources rationally in primary care settings.

## Background

Acute febrile respiratory illness (AFRI) leading to pneumonia is a common cause of morbidity and mortality throughout the world [1]. How to identify potential pneumonia cost-effectively is the priority for the physician in treating AFRI patients, because patients with pneumonia require specific anti-infection treatment. According to most clinical guidelines globally, acute pulmonary infiltrates on computerized tomography (CT) are the reference standard for diagnosing pneumonia [2–4]. However, CT scans are not always conveniently accessible or properly used in many primary care settings due to economic reasons or physicians’ clinical experience limitations. Instead, chest X-ray is used more frequently. Although chest X-ray could be an alternative in some cases, it may result in missed diagnosis of pneumonia at an early stage due to the low resolution of lung images. Especially for viral pneumonia, a little patchy interstitial infiltration at the initial stage is difficult to be identified on chest X-ray [5]. This then necessitates the need for pneumonia prediction by clinical features, which could help physicians identify potential pneumonia patients more quickly and accurately and then use CT scans to confirm diagnosis.

Several clinical features have been identified for pneumonia prediction in outpatient settings. According to a meta-analysis in adults [6], symptoms such as cough, fever, tachycardia, and dyspnea were limited as a single predictor for the diagnosis of pneumonia. Clinical features that showed the best pooled positive likelihood ratios were respiration rates > 20/min, temperature ≥ 38 °C, pulse rates > 100/min, crackles, and molecular biomarkers of procalcitonin (PCT) > 0.25 ng/ml and C-reactive protein (CRP) > 20 mg/l. However, so far clinical decision rule that combines these clinical features together for predicting radiographic pneumonia is still lacking.

In this study, we assessed the value of symptoms, signs, and laboratory tests in predicting pneumonia among AFRI patients who came to our fever clinics and worked out a simple multivariable model for pneumonia prediction in these patients. Then an external multi-center validation study was conducted with a bigger and more varied patient population in 5 primary care settings from different provinces in China.

## Methods

### Study design

This is a cohort study that was planned as a two-stage program. The first stage was to develop a simple multivariable model for pneumonia prediction in AFRI patients, and all the works were conducted in the fever clinics of Shanghai General Hospital. The second stage was to validate the model in a bigger population and with the participation of more primary care settings from different areas of China. A checklist from Transparent Reporting of a multivariable prediction model for Individual Prognosis or Diagnosis (TRIPOD) guideline was completed [7].

### Model development population

Patients who visited fever clinics in the two campuses (north campus in the urban area and south campus in the suburban area) of Shanghai General Hospital between January 22 and February 6, 2020, were enrolled in the first stage of this study. Fever clinics are primary care settings that are used to treat and triage fever patients in China. The inclusion criteria for this study were as follows: (1) patients were ≥ 18 years of age, and presented with respiratory symptoms, including running nose, nasal congestion, sore throat, cough, purulent sputum, and dyspnea; (2) body temperature ≥ 37.3℃ by ear thermometer for 2 times; (3) blood routine test and CRP were taken during the clinic visit. The biomarker procalcitonin wasn’t chosen in the study due to its unavailability for rapid measurement. Pregnant patients were excluded from the study.

Physicians, wearing protective clothing and face masks, recorded a detailed medical history and reviewed the blood test results. Once a patient had fever for more than 3 days or had epidemiological contact with novel coronavirus 2019, they ordered chest high-resolution CT scans.

### Clinical variables and predictors

Clinical characteristics of patients were collected as follows: duration of fever; accompanying symptoms; body temperatures; respiration rates; pulse rates; pulse oxygen saturation at fingertips. Results of blood tests, including CRP, were also collected. All these characteristics were included as predictors to develop a model for pneumonia prediction.

### Diagnosis

AFRI was identified in febrile patients (body temperature ≥ 37.3℃) who had one of the four respiratory symptoms including runny nose, nasal congestion, sore throat, and cough. Pneumonia was diagnosed when acute alveolar or interstitial infiltrates were found on CT images, which was interpreted independently by two experienced radiologists who were blind to the patients’ clinical characteristics. When radiologists had difficulty in determining whether infiltrates on CT images were acute or not, they excluded the related patients from the analysis.

### External validation population

An external validation population was enrolled in primary care settings from the provinces of Hubei, Jiangxi, Hainan, Guizhou, and Shanghai in China from March 1, 2020, to December 31, 2021. These participants (≥ 18 years of age) presented with fever (body temperature ≥ 37.3℃) and respiratory symptoms when visiting the clinics and agreed to undergo blood tests and CT scans. Physicians collected their data of symptoms, signs, and CRP concentrations, and the prediction model developed in the first stage was used to get a pneumonia risk mark. Patients were sent for CT scans to determine whether they had pneumonia by two radiologists independently. In patients who were diagnosed with pneumonia, pneumonia severity index (PSI) were assessed, and the numbers of infected lung lobes were collected. Sputum of these participants was cultured to clarify the bacterial pathogens, and nucleic acid tests for respiratory specimens (nasopharyngeal or oropharyngeal swab samples) were tested for viral pathogens such as SARS-COV-2. Serological tests on common respiratory pathogens (*Influenza A*, *Influenza B*, *Para-influenza*, *Mycoplasma*, *Chlamydia*,* Legionella bacteria*, *Respiratory syncytial virus*, *Adenovirus*, and *Coxsackie virus*) were also conducted. Then the performance of the prediction model was assessed for pneumonia patients with different severity and pathogens.

### Statistics

SAS software (version 8.0; SAS Institute; Cary, NC, USA) were used for statistical analysis, and graphs were drawn using GraphPad Prism (version 8; GraphPad Software, San Diego, CA, USA). A ‘Trialsize’ package of R software (version 4.0.2) was used to calculate the sample size. Complete-case analysis was used to handle missing data. Quantitative variables of normal distribution were presented as the mean ± SD. Independent *t*-test was used to determine whether normally distributed variables were different between patients with and without pneumonia. Chi-squared (*χ*^2^) test was used to determine whether qualitative variables were different between the two groups. A logistic regression model was used to determine the factors that were independently associated with pneumonia by stepwise selection. Bootstrapping method was used for internal validation. The predicted probability of pneumonia for each subject was calculated using the equation previously reported [8], and then the risk groups were created. For external validation population, a scoring system was made, with one score for each predictor item. Sensitivity and specificity statistics and receiver operating characteristic (ROC) analyses were estimated for pneumonia prediction. *Z* test was used to compare areas under curves (AUCs) between the two variables or two models by the Hanley and McNeil method. The Spearman correlation coefficient (*rs*) was used to indicate the association between the two nonparametric variables. A *P* value < 0.01 was considered to denote statistically significant differences.

## Results

### Clinical characteristics of model development population

**Fig. 1:**
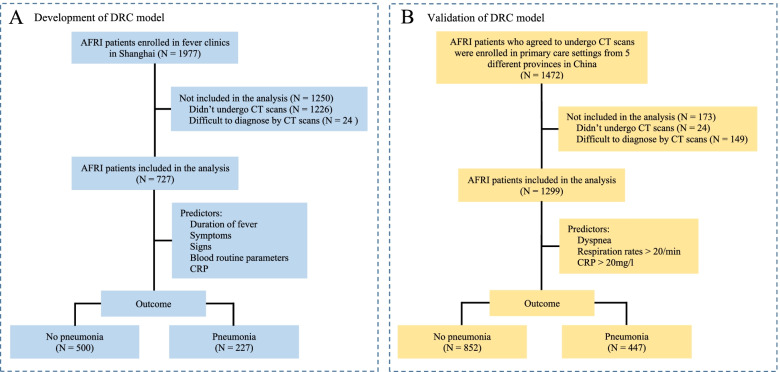
The diagram for the flow of participants through the study. **A** The development data were collected in Shanghai fever clinics between January 22 and February 6, 2020. **B** The validation data were collected in primary care settings from 5 different provinces in China between March 1, 2020, to December 31, 2021. AFRI, acute febrile respiratory illness; CT, computerized tomography

A total of 2513 patients visited fever clinics during the model development study, and 1977 of them presented with AFRI and thus were enrolled. Among these patients, 751 underwent Chest HRCT examination, and the determination of pneumonia present or absent was made in 727 patients, including 364 men and 363 women with a mean age (± SD) of 43.5 ± 17.8 years. Of these 727 patients, 227 (31.2%) had pneumonia, and 500 (68.8%) did not (Fig. 1A). AFRI patients who did not undergo CT examination (n = 1226) were treated as participants with missing data, thus were not included in the analysis. The clinical variables between patients with and without pneumonia were shown in Table 1. Compared with non-pneumonia patients, patients with pneumonia were older (*t* = 7.9, *P* < 0.0001), and had significantly longer duration of fever (*t* = 11.9, *P* < 0.0001), and higher frequencies of purulent sputum (*χ*^2^ = 93.4, *P* < 0.0001), dyspnea (*χ*^2^ = 230.0, *P* < 0.0001), and thoracic pain (*χ*^2^ = 89.0, *P* < 0.0001). Respiration rates were significantly increased in pneumonia patients (*χ*^2^ = 183.4, *P* < 0.0001). For blood parameters, higher percentages of neutrophils (*t* = 3.1, *P* = 0.0019), lower percentages of lymphocytes (*t* = 3.7, *P* = 0.0003) and eosinophils (*t* = 2.7, *P* = 0.0064), and lower hematocrits (*t* = 3.7, *P* = 0.0002) were found in pneumonia patients. CRP concentrations was significantly higher in pneumonia patients than in non-pneumonia patients (*t* = 6.9, *P* < 0.0001).

**Table 1:** Clinical characteristics and blood test results of patients with and without pneumonia in fever clinics

	No pneumonia *n* = 500	Pneumonia *n* = 227	*P*-value
Age, years (SD)	40.1 (16.2)	50.9 (19.1)	< 0.0001
Gender, male:female	1:1.07	1.18:1	0.1347
Comorbidities			
COPD,* n* (%)	20 (4.0)	11 (4.8)	0.2632
Bronchiectasis,* n* (%)	8 (1.6)	7 (3.1)	0.1922
Diabetes,* n* (%)	13 (2.6)	11 (4.8)	0.1163
Hypertension,* n* (%)	47 (9.4)	26 (11.5)	0.3932
Cardiovascular disease,* n* (%)	15 (3.0)	13 (5.7)	0.0766
Renal disease,* n* (%)	5 (1.0)	4 (1.8)	0.3891
Hepatic disease,* n* (%)	3 (0.6)	5 (2.2)	0.0549
Cancer,* n* (%)	5 (1.0)	6 (2.6)	0.0926
Symptoms			
Duration of fever, days (SD)	1.8 (0.7)	2.5 (0.8)	< 0.0001
Dry cough,* n* (%)	287 (58.4)	145 (63.9)	0.0993
Purulent sputum,* n* (%)	41 (8.2)	86 (37.9)	< 0.0001
Sore throat,* n* (%)	227 (45.4)	88 (38.8)	0.0943
Running nose/nasal congestion,* n* (%)	114 (22.8)	45 (19.8)	0.3683
Dyspnea,* n* (%)	9 (1.8)	105 (46.3)	< 0.0001
Thoracic pain,* n* (%)	136 (27.2)	146 (64.3)	< 0.0001
myalgia,* n* (%)	232 (46.4)	112 (49.3)	0.4620
Nausea,* n* (%)	149 (29.8)	49 (21.6)	0.0211
Diarrhea,* n* (%)	91 (18.2)	43 (18.9)	0.8108
Headache,* n* (%)	97 (19.4)	62 (27.3)	0.0168
Physical signs			
Fever, ℃ (SD)	38.7 (0.6)	38.8(0.6)	0.0151
Respiration rates > 20/min,* n* (%)	4 (0.8)	82 (36.1)	< 0.0001
Pulse rates, > 100/min,* n* (%)	352 (70.4)	168 (74.0)	0.3177
Pulse SaO2%, (SD)	98.1 (1.8)	97.0 (3.2)	0.1980
Blood parameters			
Red blood cell, × 10^12^/L (SD)	4.6 (0.5)	4.5 (0.7)	0.0574
Hemoglobin, g/L (SD)	139.3 (17.3)	136.3 (19.7)	0.0510
White blood cell, × 10^9^/L (SD)	8.1 (3.6)	8.4 (3.9)	0.1507
Neutrophils, × 10^9^/L (SD)	5.7 (3.3)	6.2 (3.6)	0.0675
Lymphocytes, × 10^9^/L (SD)	1.6 (0.8)	1.5 (0.8)	0.0225
Monocytes, × 10^9^/L (SD)	0.5 (0.3)	0.6 (0.3)	0.0101
Eosinophils, × 10^9^/L (SD)	0.1 (0.1)	0.1 (0.1)	0.0566
Basophils, × 10^9^/L (SD)	0.0 (0.1)	0.0 (0.1)	0.5344
Thrombocyte, × 10^9^/L (SD)	163.9 (55.8)	162.0 (59.8)	0.6621
Neutrophils %, (SD)	68.5 (12.4)	71.6 (12.7)	0.0019
Lymphocytes %, (SD)	22.7 (10.7)	19.6 (10.6)	0.0003
Monocytes%, (SD)	7.2 (3.3)	7.6 (3.3)	0.1823
Eosinophils%, (SD)	1.2 (1.4)	0.9 (1.2)	0.0064
Basophils%, (SD)	0.3 (0.6)	0.3 (0.6)	0.4440
Hematocrit%, (SD)	43.4 (4.9)	41.9 (5.5)	0.0002
C-reactive protein, mg/l (SD)	17.7 (35.5)	42.1 (56.8)	< 0.0001

### Development of a model for pneumonia prediction

**Fig. 2:**
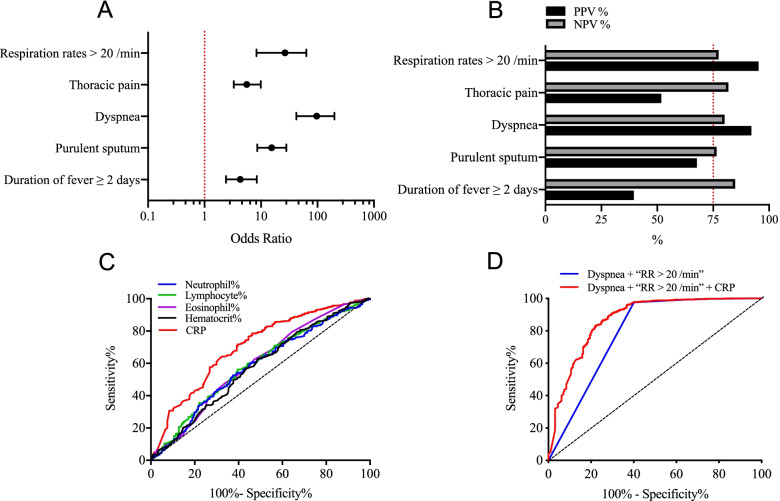
Development of a clinical model for pneumonia prediction in acute febrile patients. The odd ratios (**A**) and predictive values (**B**) of clinical characteristics were calculated for the prediction of pneumonia. Dyspnea and respiration rates > 20/min were two predictors that showed high odd ratios and had good performance on both PPV and NPV. **C** ROC curves for pneumonia prediction were analyzed among blood parameters. In these parameters, CRP yielded significantly higher accuracy than other parameters, with an AUC of 0.7249. **D** Predictive values of “Dyspnea + RR > 20/min” model and “Dyspnea + RR > 20/min + CRP” model were compared for pneumonia. The addition of CRP significantly improved the AUC from 0.7900 to 0.8716 (*P* < 0.01). CRP, C-reactive protein; RR, respiration rates; ROC, receiver operating characteristic; AUC, area under the curve; PPV, positive predictive value; NPV, negative predictive value

Figure 2A showed the ORs of five selected variables (duration of fever ≥ 2 days, purulent sputum, dyspnea, thoracic pain, and respiration rates > 20/min) from symptoms and signs that were significantly more frequent in pneumonia patients. ORs were statistically significant in all these five variables, with dyspnea showing the highest value of 97.0 (95% CI, 42.3–199.4). Positive predictive value (PPV) and negative predictive value (NPV) for these variables were calculated (Fig. 2B). Dyspnea and respiration rates > 20/min showed good predictive value in both PPV and NPV. Although duration of fever ≥ 2 days, purulent sputum and thoracic pain had good NPVs, their PPVs were relatively poor, resulting in significantly lower accuracy than that of dyspnea and respiration rates > 20/min (*P* < 0.01), thus were not selected for further analysis.

Predictive performance for pneumonia was analyzed in five selected blood parameters (percentage counts of neutrophils, lymphocytes, and eosinophils, percentages of hematocrit, and serum concentrations of CRP) that were significantly different between pneumonia and non-pneumonia patients. Corresponding ROC curves for these parameters were constructed in Fig. 2C. In these parameters, CRP yielded significantly higher accuracy than other parameters (*Z* = 3.3, *P* < 0.01), with an AUC of 0.7249 (95% CI, 0.7019–0.7433).

The model development had several steps. Firstly, logistic regression analysis was performed with symptom (dyspnea) and sign (respiration rates > 20/min) for pneumonia prediction (227 pneumonia and 500 non-pneumonia). Both variables had statistically significant ORs, and the “symptom and sign” model was constructed as follows.

$$\mathrm{ln}[P/1-P] = -1.67+3.47\times D+3.74\times R.$$

*P* = predicted probability of pneumonia. *D* = dyspnea (yes, 1; no, 0). *R* = respiration rates (> 20 /min, 1; ≤ 20/min, 0).

Then, the predicted probability of pneumonia for individual patients was calculated according to the model, and its predictive value was analyzed using ROC curves, yielding an AUC of 0.7900 (95% CI, 0.7487–0.8313). When CRP was added, as a continuous variable, to the logistic regression analysis as follows, the predictive value of “symptom and sign” model was significantly improved (*Z* = 2.7, *P* < 0.01), with an AUC of 0.8716 (95% CI, 0.8409–0.9023) (Fig. 2D).

$$\mathrm{ln}[P/1-P] =-1.98+3.47\times D+3.54\times R+0.01\times C.$$

*P* = predicted probability of pneumonia. *D* = dyspnea (yes, 1; no, 0). *R* = respiration rates (> 20/min, 1; ≤ 20 /min, 0). *C* = CRP (mg/l).

ORs and predictive performance for five clinically relevant cut-off values of CRP (> 10 mg/l, > 20 mg/l, > 30 mg/l, > 40 mg/l, and > 50 mg/l) were calculated in the “symptom and sign + CRP” model. All ORs were statistically significant for different CRP cutoffs. When CRP > 20 mg/l was chosen for the model, the AUC reached the highest value of 0.8506 (95% CI, 0.8158–0.8854), thus CRP > 20 mg/l was included in the final model as follows.

$$\mathrm{ln}[P/1-P] = -2.12+3.46\times D+3.56\times R+1.35\times C.$$

*P* = predicted probability of pneumonia. *D* = dyspnea (yes, 1; no, 0). *R* = respiration rates (> 20/min, 1; ≤ 20 /min, 0). *C* = CRP (> 20 mg/l, 1; ≤ 20 mg/l, 0).

For the convenience of the model application among AFRI patients in primary care settings, a scoring system was made with one score for each item. Based on the DRC model, the high-risk group for pneumonia consisted of patients positive for all three items (3 scores). The combined probability value in this high-risk group of having pneumonia was 99.8% (95% CI = 99.6–99.9%). Moderate-risk group for pneumonia consisted of patients positive for one or two of these items (1–2 scores), and the combined probability value was 59.8% (95% CI = 56.2–65.3%). Low-risk group for pneumonia consisted of patients without positive score (0 score) on the three items, and the combined probability value was 9.7% (95% CI = 9.3%-10.5%).

Setting the criteria of ≥ 1 score as the cut-off point, the sensitivity, and specificity of the DRC model for pneumonia prediction in the model development population (*n* = 727) was 81.1% and 80.2%, respectively. Notably, in patients whose age was above 60 years (*n* = 159), its sensitivity and specificity were 84.0% and 84.6%, suggesting the model had good predictive capacity for old patients. If the prediction model was applied to the patients who received CT examination, CT could have been avoided in 401 (55.2%) of these patients, with a risk of missing 43 patients (5.9%) with pneumonia. For the 43 patients mispredicted by the model, all of them had at least one of other risk factors including duration of fever ≥ 2 days, purulent sputum, and thoracic pain.

### External validation study of the DRC model

We assessed the predictive value of the DRC model in an external validation population than included 1472 AFRI patients in primary care settings from 5 different provinces in China. Among these patients, 1299 patients (681 men and 618 women with a mean age (± SD) of 42.4 ± 17.9 years) were included in the analysis (Fig. 1B). HRCT scans determined that 447 patients had pneumonia and the other 852 patients didn’t. For patients who had pneumonia, respiratory pathogens could be identified in 308 patients, including 93 patients with bacterial pathogens, 171 patients with viral pathogens, 41 patients with atypical pathogens, and 3 patients with fungal pathogens. No significant difference was found in the clinical characteristics and blood test results between development population and validation population.

**Fig. 3:**
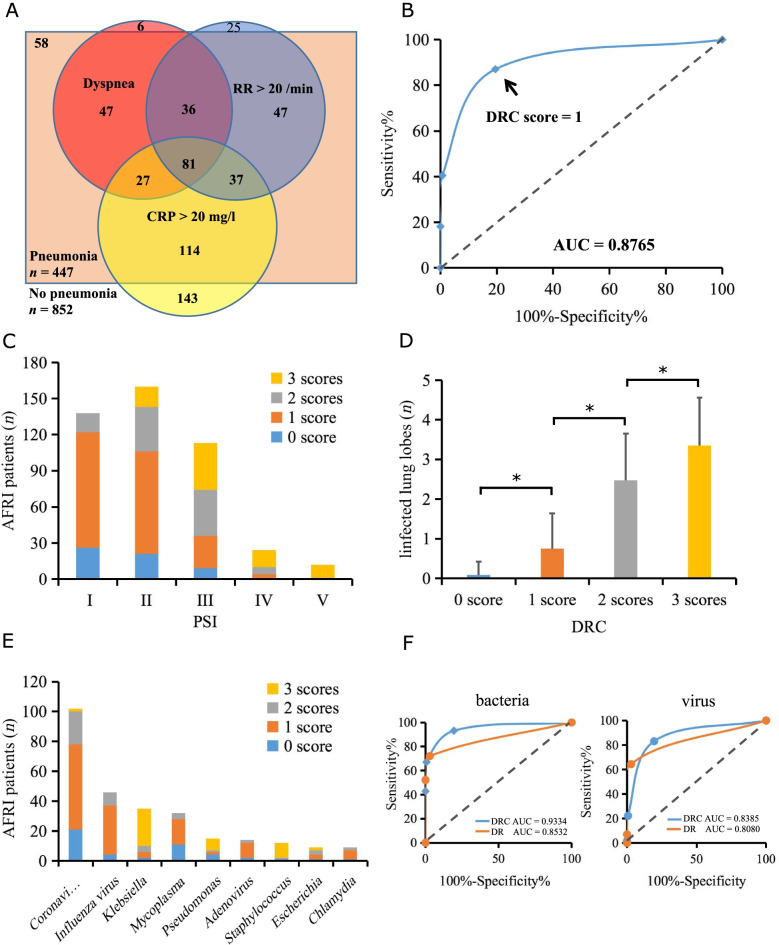
The value of DRC model (dyspnea, respiration rates > 20/min, and C-reactive protein > 20 mg/l) for pneumonia prediction in external validation population. **A** Venn diagrams showed the overlaps among patients with dyspnea, respiration rates > 20/min, and C-reactive protein > 20 mg/l. **B** ROC curve of DRC model showed the predictive accuracy was highest when choosing at least one positive item (1 score) as cut-off point. **C** The numbers of AFRI patients with different DRC scores were summarized in different pneumonia-severity groups. The average DRC scores in each severity group increased with the elevation of patients’ PSI classes. **D** The numbers of infected lung lobes increased with the rise of DRC scores. Data were presented as mean ± SD. *P < 0.01. **E** The numbers of AFRI patients with different DRC scores were summarized according to different respiratory pathogens. **F** ROC curves of DRC model and its simplified form, DR model (dyspnea and respiration rates > 20/min), for pneumonia prediction. For bacterial pneumonia, the AUC of the DR model was significantly less than that of the DRC model (*P* < 0.01). However, for viral pneumonia, no significant difference was found in the AUCs between two models. RR, respiration rates; CRP, C-reactive protein; ROC, receiver operating characteristic; AFRI, acute febrile respiratory illness; PSI, pneumonia severity index; AUC, area under curve

The scores of the DRC model were calculated and analyzed as shown in Fig. 3. Dyspnea was present in 191 (42.7%) pneumonia patients, respiration rates > 20/min was present in 201 (45.0%) pneumonia patients, and CRP > 20 mg/l was present in 259 (57.9%) pneumonia patients. Overlaps among these patients were shown in Fig. 3A. ROC analysis indicated that DRC model had good performance for pneumonia prediction with an AUC of 0.8765 (95% CI, 0.8549–0.8980), and the predictive accuracy was the highest when choosing at least one positive item (1 score) as cut-off point (sensitivity = 87.0%, specificity = 80.5%, Fig. 3B).

PSI scores were assessed for pneumonia patients, and DRC scores were analyzed in different PSI classes. We found DRC scores showed a positive correlation with PSI scores (*rs* = 0.5020, *P* < 0.0001). In PSI class V, the percentage of patients who had 3 scores of the DRC model reached 91.7% (Fig. 3C). DRC scores also showed a positive correlation with the numbers of infected lung lobes (*rs* = 0.6511, *P* < 0.0001). For patients who got 3 scores, the number of lobes reached 3.3 ± 1.2 (Fig. 3D).

Among patients with detected respiratory pathogens, DRC scores were calculated according to their different infecting pathogen genus (Fig. 3E). As a whole, DRC scores were higher in those with bacterial pathogens (2.0 ± 1.0) than those with viral ones (1.1 ± 0.7, *P* < 0.01). ROC curve was analyzed for pneumonia prediction with bacterial or viral pathogens. We found the model showed good predictive performance for both bacterial and viral pneumonia, with AUC of 0.9334 and 0.8385 respectively (Fig. 3F). We also analyzed the difference in ROC curves between the DRC model and its simplified form DR model (composed of dyspnea and respiration rates > 20/min only) in different pathogen groups. For bacterial pneumonia, the AUC of the DR model was significantly less than that of the DRC model (*Z* = 3.2, *P* = 0.0016). However, for viral pneumonia, no significant difference was found in the AUCs between the two models (*Z* = 1.0, *P* = 0.2993), suggesting dyspnea plus respiration rates > 20/min had a good predictive capacity for viral pneumonia even without the result of CRP.

## Discussion

Pneumonia prediction in AFRI patients can facilitate the early diagnosis and prompt treatment of pneumonia, which is particularly important for patients in primary care settings where CT scans are not frequently or routinely used due to economic reasons or clinical issues. In this study, we analyzed the difference of clinical features between pneumonia and non-pneumonia among AFRI patients in fever clinics and developed a DRC model comprised of three items, including dyspnea, respiration rates > 20 /min, and CRP > 20 mg/l, for pneumonia prediction. In the external multi-center validation population from primary care settings in 5 different provinces around China, this model was confirmed to have good performance for pneumonia prediction with an AUC of 0.8765. DRC scores increased with pneumonia severity and lung lobe involvement and showed good predictive accuracy in both bacterial and viral pneumonia. For viral pneumonia, dyspnea plus respiration rates > 20/min had good predictive capacity even without the result of CRP concentration.

Our results showed that dyspnea and respiration rates > 20/min were the two most valuable predictors for pneumonia in fever clinics. Dyspnea is caused by abnormal ventilation and gas exchange in the presence of interstitial and alveolar edema. Although it may well be present in patients with bacterial pneumonia, it seems that patients with viral pneumonia are more likely to have dyspnea when multiple lobes are involved [9]. Respiration rates > 20/min is an objective sign that reflects airway resistance caused by pneumonia and is correlated with the severity of dyspnea [10]. Our results showed that the combination of the two predictors had good value for pneumonia prediction, which is consistent with the pathophysiological characteristics of pneumonia.

Blood routine test is the common laboratory test that physicians use to identify pneumonia. However, in this study, we did not find any difference in the absolute counts of blood cells between pneumonia and non-pneumonia patients. This seeming discrepancy could be related to the various effects on blood cell counts by different respiratory pathogens [11]. For example regarding viral pathogens, SARS-COV-2 is known to induce lymphopenia in peripheral blood, but other viral pathogens have been reported to induce a higher lymphocyte counts [12]. Although the percentage counts of blood cells had clinical significance for pneumonia, their performances were not good enough for pneumonia prediction according to our ROC data. Therefore, it is difficult to identify pneumonia based on blood routine tests.

Elevated CRP is another important index for pneumonia. Several studies have showed CRP had a significant advantage in the diagnosis of pneumonia among patients with acute cough or lower respiratory tract infection [8, 13, 14]. The good value of CRP for pneumonia prediction shown in our AFRI patients is consistent with previous reports. When combined with dyspnea and respiration rates > 20/min, its predictive value was even higher, indicating the advantage of the DRC model over CRP alone. However, previous studies indicated that CRP had insufficient sensitivity and specificity in predicting a viral cause [15, 16]. This is consistent with our validation results that dyspnea plus respiration rates > 20/min had good predictive capacity for viral pneumonia even without the result of CRP concentration.

For the convenience of the application of the DRC model among AFRI patients in primary care settings, a scoring system was made, with one score for each item. When a patient gets 3 scores, his/her risk of pneumonia is high. In this case, CT scan is strongly recommended for the confirmation of pneumonia. When a patient gets 1–2 score, his/her risk of pneumonia is moderate, and CT scan should be considered, especially for those who have a contact history of epidemic pathogens. When a patient gets 0 score, his/her risk of pneumonia is low, and CT scan is not necessary unless other risk factors were present, including duration of fever ≥ 2 days, purulent sputum, and thoracic pain. Our validation results showed that all the patients who got 2 or 3 scores were confirmed to have pneumonia by CT scans, and 92.1% of patients who got 0 score did not have pneumonia according to their CT images.

In the external multi-center validation population, DRC scores showed a positive correlation with PSI scores and numbers of infected lung lobes for pneumonia patients in primary care settings, and thus it might facilitate the assessment of disease severity and the rational allocation of medical resources. In addition, our results showed that dyspnea plus respiration rates > 20/min had a good predictive capacity for viral pneumonia regardless of CRP concentration. So in the epidemic of coronavirus disease 2019, AFRI patients who have dyspnea or respiration rates > 20/min are suggested to undergo CT scans even without the result of CRP concentration [17].

One limitation of our study was that we did not investigate the impact of comorbidity medications on the predictive capacity of the DRC model, so attention should be paid when applying the DRC model to AFRI patients who had comorbidities. Further studies are still needed.

## Conclusions

DRC model is a simple tool for pneumonia prediction that is composed of items on dyspnea, respiration rates > 20 /min, and CRP > 20 mg/l. In the external validation study, it showed good predictive performance among AFRI patients in primary care settings. In future application, the model could help physicians apply CT scans to AFRI patients of moderate-to-high pneumonia risks, which would be particularly helpful for the triage of patients in primary care settings.

## Data Availability

The data that support the findings of this study are available from Min Zhang upon reasonable request.

## Abbreviations

AFRI: Acute febrile respiratory illness
AUC: Area under curve
CRP: C-reactive protein
CT: Computerized tomography
DRC: Dyspnea, respiration rates > 20/min and CRP > 20 mg/l
PPV: Positive predictive value
NPV: Negative predictive value
RR: Respiration rates
PSI: Pneumonia severity index
ROC: Receiver operating characteristic
